# Investigating the Prevalence and Associated Factors of Anxiety and Depressive Symptoms Among Pregnant Women in Bangladesh: A Hospital‐Based Cross‐Sectional Study

**DOI:** 10.1002/hsr2.71144

**Published:** 2025-08-06

**Authors:** Noushin Rahman Mahin, Md Jamil Hossain, Bishwjit Bhowmick, Lakshmi Rani Kundu

**Affiliations:** ^1^ Department of Public Health and Informatics Jahangirnagar University Savar Dhaka Bangladesh; ^2^ Department of Biochemistry and Molecular Biology Jahangirnagar University Savar Dhaka Bangladesh

**Keywords:** anxiety, Bangladesh, depression, perinatal anxiety, perinatal depressive symptoms, pregnant women

## Abstract

**Background:**

Anxiety and depression during pregnancy are significant public health concerns in low‐ and middle‐income countries like Bangladesh, with potential adverse effects on both mothers and infants. However, there is limited research on prenatal mental health in Bangladesh. This study aims to investigate the prevalence and associated factors of anxiety and depressive symptoms among pregnant women.

**Methods:**

A cross‐sectional study was conducted using systematic random samples to recruit participants from a hospital in Dhaka. A total of 227 pregnant women were interviewed with a structured interview, including sociodemographic characteristics, physiological condition, family support, intimate partner violence, obstetric history, and mental health outcomes. Depression and anxiety were measured using the Edinburgh Postnatal Depression Scale (EPDS) and the State‐Trait Anxiety Inventory (STAI). The *χ* ^2^ tests and multiple logistic regression were used to identify associated factors. The data were analyzed using SPSS software version 26.

**Results:**

Among the 227 participants, 25.1% reported depressive symptoms and 35.7% reported anxiety symptoms. Significant associations were observed between depression and factors such as occupation, relationship with husband, and forced sex. Additionally, anxiety was significantly associated with gestational age. Regression analysis revealed that housewives were less likely to experience depression (AOR = 0.17 [95% CI: 0.05–0.55], *p*‐value = 0.003), while participants who experienced forceful sex were at higher risk (AOR = 4.39 [95% CI: 0.97–19.82], *p*‐value = 0.0542). Anxiety symptoms were more likely among women in the first trimester (AOR = 2.63, 95% CI: 1.27–5.45, *p*‐value = 0.028) and second trimester (AOR = 1.42 [95% CI: 0.71–2.84], *p*‐value = 0.028), compared to those in the third trimester.

**Conclusions:**

Anxiety and depression are prevalent among pregnant women in Bangladesh. Provide special attention and practical support to working women and women in their first and second trimesters when adopting mental health initiatives.

**Abbreviations**
AOR: adjusted odds ratio
BMI: body mass index
CAD: comorbid anxiety and depression
CI: confidence Interval
EPDS: the edinburgh postnatal depression scale
LBW: low birth weight
STAI: the state‐trait anxiety inventory

## 1 Introduction

Women experience some physical, mental, and social transitions during pregnancy, and try to adjust to these changes [1]. Pregnancy and childbirth are life‐changing experiences for women [2]. Importantly, unpreparedness for pregnancy and the impact of pregnancy hormones make a woman more vulnerable to physical and mental changes [3]. However, during this phase, there is a rise in mental health problems, including anxiety and depression [4]. Even among mentally stable women, pregnancy can create a lot of anxiety due to the unknown. Stress and anxiety over childbirth differ from one woman to the next [5]. Mental health problems like depression and anxiety have become a serious public health threat because of their high incidence rate in pregnancy [5]. Around 24.7% of pregnant women experience mental health problems, predominantly depression, in low‐income countries [6]. These psychological and social changes cause a wide range of adverse effects the pregnant women, their offspring, and their families [7].

During pregnancy, some women may get their first depressive episode [8]. Several factors, such as older age, lower levels of education, marital problems, excessive household loads, and pregnancy symptoms, were strongly connected with depression [9]. Premji et al. have discovered that individuals who have a high degree of perceived stress, have more than three children, and have had bad childhood experiences are more likely to develop depression and anxiety [10]. Additionally, the women who suffered from domestic violence were found to be more likely to have anxiety and depression [11]. Similarly, partner abuse, not supportive partners, was an important factor in antenatal depression [12]. However, depression during pregnancy was connected with poor birth outcomes [13], including early deliveries, low birth weight (LBW), and poor growth of infants [14, 15]. Similarly, antenatal depression and anxiety result in postnatal depression [16]. In addition, it causes birth asphyxia, coronary heart disease, and poor cognitive development in infants [17, 18, 19]. Furthermore, comorbid anxiety and depression (CAD) also increase the frequency of prolonged labor and delayed beginning of breastfeeding [20].

According to a systematic review and meta‐analysis, in developing countries, depression is considered one of the most prevalent psychological problems in pregnancy, affecting around 19.2%–34% of pregnant women, which varies in terms of their sociodemographic characteristics and economic status [6, 21, 22, 23]. A study on South Indian pregnant women reported that 21.98% of women suffer from depression at the time of ANC visits [24]. According to a study on Pakistan, the prevalence rate of depression during pregnancy was 37% [25]. Regarding a comprehensive analysis, the overall prevalence of anxiety and depression during pregnancy in Africa was 15.2% and 27%, respectively [26]. In China, the prevalence of anxiety and depression symptoms was estimated to be 11.1% and 10.3% [27]. In Bangladesh, the prevalence of antenatal depression symptoms (19.5%) and anxiety symptoms (28.8%) [28, 29]. In rural areas of Bangladesh, there was a higher‐than‐average prevalence of antenatal depression symptoms (56%) [30]. The existing literature lacks comprehensive data on the prevalence of depression and anxiety among pregnant women in hospital settings in Bangladesh.

This study seeks to fill the knowledge gap regarding the prevalence and determinants of anxiety and depressive symptoms among pregnant women in hospital settings in Bangladesh. The findings of this study can guide the development of targeted interventions and support services to improve the mental health outcomes of pregnant women and their offspring. Moreover, this study aims to contribute to the formulation of effective public health strategies for the prevention and management of depression and anxiety during pregnancy.

## 2 Methods

### 2.1 Study Design and Participants

This cross‐sectional study conducted from August 15, 2024, to October 22, 2024, among pregnant women attending maternity care in a government hospital named the Institute of Child and Mother Health, Matuail, Dhaka, Bangladesh. To participate in this study, participants had to meet the following criteria: (i) Participants must be pregnant women, (ii) participants had to attend a maternity government hospital, (iii) participants had to visited the hospital during the data collection period, (iv) the participants had the willingness to participate in the study voluntarily. Exclusion criteria: (i) prior diagnosis of mental illness, (ii) intellectual disability, (iii) neurodevelopmental disorders, and (iv) any severe physical illness that might impair their ability to participate in the study. A systematic random sampling technique was adopted based on their serial number to select the participants.

### 2.2 Sample Size Determination

The following formula was used for the determination of sample size:

$$n=\frac{z^{2}\mathrm{pq}}{d^{2}};\;n=\frac{1.96^{2}\times 0.18\times \left(1-0.18\right)}{0.05^{2}}=226.81\approx 227$$

Where *z* = 1.96 at 5% level of significance; *d *= margin of error taken as 5%; *p *= the prevalence of depression found from a previous study, 18%; *q* = 1−*p* = 0.82. Therefore, the minimum required sample size calculated for this study was 227.

We initially approached 247 eligible pregnant women during the study, out of which 235 agreed to participate (non‐response rate: 5%). Among the 235 questionnaires, eight responses were excluded due to missing or inconsistent data. As a result, a final sample of 227 valid data points was included in the final analysis.

### 2.3 Data Collection Tools and Procedure

Data was collected using a modified version of validated questionnaires used in existing studies [20] through face‐to‐face interviews. The questionnaire is divided into seven modules, which include sociodemographic questions, followed by modules on physiological conditions, social support, intimate partner violence, obstetric history, and finally, anxiety and depressive symptoms. The structured questionnaire was initially prepared in English and then translated into Bangla, the native language. We utilized the Bangla version of the questionnaire for data collection.

Five enumerators, comprising public health students, were initially selected for data collection and received a 5‐day offline training by a faculty member from the Department of Public Health and Informatics at Jahangirnagar University, Savar, Dhaka‐1342. The training included familiarization with the study objectives, ethical considerations, and guidelines for conducting interviews to ensure consistency and reliability in the data collection process. The interviewers ensured that participants fully understood the questions, particularly those with limited literacy.

### 2.4 Consent Procedures

An informed consent form was prepared in English and Bengali to ensure clarity. It outlined the study's objectives, procedures, potential risks, benefits, confidentiality measures, and the voluntary nature of participation. Participants were informed of their right to withdraw at any time without consequences, and it was emphasized that their information would only be used for this study. After addressing any questions, enumerators confirmed the participants' willingness to participate and obtained signed consent. For illiterate participants, enumerators explained the form verbally and obtained verbal consent in the presence of a witness, with appropriate documentation to ensure transparency.

## 3 Measures

### 3.1 Sociodemographic Measure

Sociodemographic information was collected through closed‐ended questionnaires, including age, educational qualification, husband's education, occupation, husband's occupation, monthly family income, type of family (categorized as nuclear and joint family) [31], and place of residence.

### 3.2 Physiological Condition

Participants' physiological condition was measured based on height, weight, BMI (categorized as Underweight = BMI < 18.5, Normal weight = BMI 18.5–24.9, Overweight = BMI ≥ 25.0–29.9), Obesity = BMI ≥ 30.0) as per WHO guidelines [32], presence of anemia, gestational age, and the age at first marriage.

### 3.3 Social and Family Support

Social and family support was categorized by overall relationships with the husband and mother‐in‐law. Practical support from family and society includes any kind of domestic help with household chores.

### 3.4 Spouse Violence

Physical abuse in the past, forced sex in the past, and physical violence during pregnancy were all examples of intimate partner violence.

### 3.5 Obstetric History

The number of existing and dead children, the desired current pregnancy, history of abortion, and antenatal counseling by health personnel were all obstetric characteristics.

### 3.6 Depression

Depressive symptoms were assessed using the Edinburgh Postnatal Depression Scale (EPDS) [33]. The EPDS is a 10‐item scale whose score ranges from 0 to 30 (0 = As much as I always could to, 3 = Not at all), where a higher score indicates more depressive symptoms. The scale was previously validated in Bangladesh and detected a sensitivity and specificity of about 89% and 87%, respectively [34]. A cut‐off of ≥ 10 was used to detect depression, and less than that was considered as an absence of depression among the participants. This scale was also employed in earlier Bangladeshi studies [35].

### 3.7 Anxiety

Anxiety was assessed using the trait‐anxiety scale of the State‐Trait Anxiety Inventory (STAI). The scale is frequently used to assess anxiety during pregnancy [36]. It contains 20 items, with scores ranging from 20 to 80 (where 1 = Never and 4 = Always). A cut‐off score ≥ 45 was used to detect anxiety [37]. This scale was also used in the previous research in Bangladesh [35].

## 4 Statistical Analysis

Descriptive statistics, such as frequency and percentage, were used to report categorical variables. Additionally, continuous variables were presented as mean and standard deviation (SD). The *χ* ^2^ test was conducted to investigate the association between depression and anxiety with socio‐demographic variables, family support, spouse violence, obstetric history, and physiological condition. Additionally, a multiple logistic regression model was used to identify the risk factors associated with depression and anxiety. The regression coefficients obtained from the model were presented as adjusted odds ratios (AOR) along with 95% confidence intervals (CI). A *p*‐value < 0.05 (two‐sided) was considered statistically significant for all types of analysis. The data was analyzed using IBM SPSS Statistics (version 26). This study followed the Statistical Analyses and Methods in the SAMPL guidelines in statistical procedures [38].

### 4.1 Ethical Statement

The study was approved by the Biosafety, Biosecurity, and Ethical Review Board of Jahangirnagar University, Savar, Dhaka‐1342, Bangladesh (Ref. No: BBEC, JU/M 2024/08 (123)). Additionally, another permission was obtained from the authority of the Institute of Child and Mother Health, Matuail, Dhaka, before data collection. Participants were informed about the nature and purpose of the study and the confidentiality of their data. They had the right to withdraw from the study at any time during the interview. Additionally, informed written consent was obtained from all participants. It has been confirmed that all experiments were performed following relevant guidelines and regulations.

## 5 Results

### 5.1 Sociodemographic Information of the Participants

Table 1 shows the sociodemographic characteristics of the participants. In a total of 227 pregnant women, the majority (92.5%) of the respondents' age group was between 19 and 34 years. The mean age of the participants was 24.29 (SD = 4.4). Almost 40.5% of women had finished their higher education, and 46.3% of women′s husbands had higher education. Most of the individuals (91.6%) were housewives, while 42.3% of their husbands were jobholders. In terms of income, the largest proportion of respondents (44.9%) reported a monthly household income of less than 20,000 BDT (1 BDT = $0.0082 as of May 20, 2025), and over half of the respondents lived in a nuclear family (59.5%). Furthermore, a significant majority (81.5%) lived in urban areas.

**Table 1:** Sociodemographic characteristics of the study participants (*N *= 227).

Variable	Categories	Frequency (*n*)	Percentage (%)
Age of the participants (years)			24.29 (SD = 4.4)
Age group	≤ 18	10	4.4
	19–34	210	92.5
	≥ 35	7	3.1
Educational qualification	Illiterate	5	2.2
	Primary	70	30.8
	Secondary	60	26.4
	Higher	92	40.5
Husband's educational qualification	Illiterate	15	6.6
	Primary	58	25.6
	Secondary	49	21.6
	Higher	105	46.3
Occupation	Housewife	208	91.6
	Working woman	19	8.4
Husband's occupation	Teacher	5	2.2
	Job holder	96	42.3
	Businessman	52	22.9
	Day laborer	8	3.5
	Others	66	29.1
Monthly income	< 20,000 BDT (< 163.93 USD)	102	44.9
	20,000–40,000 BDT (163.93–327.86 USD)	79	34.8
	> 40,000 BDT (> 327.86 USD)	46	20.3
Family type	Nuclear	135	59.5
	Joint	92	40.5
Place of residence	Urban	185	81.5
	Rural	42	18.5

Abbreviations: BDT = Bangladeshi Taka, USD = United States Dollar.

### 5.2 Physiological Condition, Obstetric History, Family Support, and Husbands' Violence‐Related Information

Table 2 demonstrates the physiological condition, obstetric information, family support, and husbands' violence among pregnant women. The majority of pregnant women (62.9%) had a healthy weight (BMI: 18.5–24.9), while 18.5% were overweight (BMI: 25–29.9), 9.3% were underweight (BMI: < 18.5), and 8.8% were obese (BMI: ≥ 30). Regarding pregnancy stages, the majority of pregnant women, 38.3% were in their second trimester, followed by 32.6% in the third trimester and 29.1% in the first trimester. Additionally, 75.3% of pregnant women had no history of anemia. However, the majority of pregnancies (79.7%) claimed to have been planned, whereas 20.3% had unplanned pregnancies. Among the participants, 58.1% were multipara, and 86.8% had no history of abortion. Regarding spousal violence, 83.3% of pregnant women never experienced physical violence by their husbands, and the majority (96%) of them never faced violence during pregnancy. Only 3.5% of women experienced forced sex in their married lives.

**Table 2:** Physiological condition, obstetric information, family support, and husband's violence among pregnant women.

Components	Variables	Categories	Frequency (*n*)	Percentage (%)
Physiological condition	Gestational age (in weeks)	First trimester (0–13 weeks)	66	29.1
		Second trimester (14–27 weeks)	87	38.3
		Third trimester (28–42 weeks)	74	32.6
	BMI	Under weight	21	9.3
		Healthy weight	142	62.6
		Overweight	42	18.5
		Obese	20	8.8
	Anemia	Yes	56	24.7
		No	171	75.3
Obstetric information	Parity	Primipara	95	41.9
		multipara	132	58.1
	History of abortion	Yes	30	13.2
		No	197	86.8
	Planed pregnancy	Yes	181	79.7
		No	46	20.3
Family support	Relationship with husband	Good	219	96.5
		In‐between	7	3.1
		Bad	1	0.4
	Relationship with mother‐in‐law	Good	187	82.4
		In‐between	7	3.1
		Bad	8	3.5
		Not applicable (e.g., if dead)	25	11.0
	Received help in daily activities	Yes	198	87.2
		No	29	12.8
Husbands' violence	Faced physical violence	Yes	38	16.7
		No	189	83.3
	Faced physical violence during pregnancy	Yes	9	4.0
		No	218	96.0
	Faced forced sex	Yes	8	3.5
		No	219	96.5

### 5.3 Prevalence of Anxiety and Depression During Pregnancy

**Figure 1:**
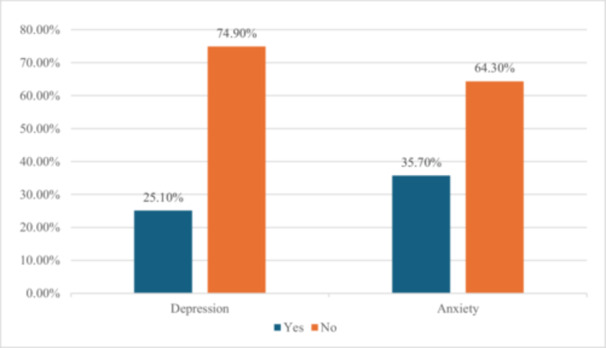
Prevalence of depressive and anxiety symptoms among pregnant women (*N* = 227).

Out of the 227 participants, 57 pregnant women (25.1%) (95% CI: 19.8–30.4) had an EPDS score of ≥ 10. The mean EPDS score among the participants was 6.72 (SD = 4.94). Similarly, 81 respondents (35.7%) (95% CI: 29.1–41.8) screened positive for anxiety, with an STAI score of ≥ 45. The mean STAI score for anxiety was 46.26 (SD = 4.82) (Figure 1).

### 5.4 Association Between Depression and Anxiety and Predictors During Pregnancy

Table 3 illustrates the association between depression and anxiety and their predictors during pregnancy. The occupation of pregnant women demonstrated a statistically significant relationship with depression (*p* = 0.004), with the majority (82.5%) of depressed women being housewives. Moreover, gestational age (in weeks) showed a significant association with anxiety (*p* = 0.025). Pregnant women in their first trimester had a higher prevalence of anxiety cases compared to those in later stages of pregnancy. Additionally, the quality of the relationship with husbands showed a strong association with depression (*p* < 0.001). A substantial proportion of participants (87.7%) who had good relationships with their husbands were free from depression. Moreover, women who experienced forced sex showed a significant association with depression (*p* = 0.013).

**Table 3:** Association between depression and anxiety and predictors during pregnancy.

Variable	Categories	Depression		*p*‐value	Anxiety		*p*‐value
		No *n* (%)	Yes *n* (%)		No *n* (%)	yes *n* (%)	
Age	≤ 18	8 (4.7)	2 (3.5)	0.133	5 (3.4)	5 (6.2)	0.569
	19–34	159 (93.5)	51 (89.5)		137 (93.8)	73 (90.1)	
	≥ 35	3 (1.8)	4 (7.0)		4 (2.7)	3 (3.7)	
Educational qualification	Illiterate	4 (2.4)	1 (1.8)	0.589	5 (3.4)	0 (0)	0.108
	primary	56 (32.9)	14 (24.6)		50 (34.2)	20 (27.7)	
	Secondary	45 (26.5)	15 (26.3)		34 (23.3)	26 (32.1)	
	Higher	65 (38.2)	27 (47.4)		57 (39.0)	35 (43.2)	
Husband's educational qualification	Illiterate	12 (7.1)	3 (5.3)	0.749	10 (6.8)	5 (6.2)	0.158
	primary	46 (27.1)	12 (21.1)		44 (30.1)	14 (17.3)	
	Secondary	36 (21.1)	13 (22.8)		31 (21.2)	18 (22.2)	
	Higher	76 (44.7)	29 (50.9)		61 (41.8)	44 (54.3)	
Occupation	Housewife	161 (94.7)	47 (82.5)	**0.004**	134 (91.8)	74 (91.4)	0.912
	Working women	9 (5.3)	10 (17.5)		12 (8.2)	7 (8.6)	
Husband's occupation	Teacher	5 (2.9)	0 (0)	0.313	4 (2.7)	1 (1.2)	0.891
	Job holder	66 (38.8)	30 (52.6)		61 (41.8)	35 (43.2)	
	Businessman	42 (24.7)	10 (17.5)		34 (23.3)	18 (22.2)	
	Day laborer	6 (3.5)	2 (3.5)		6 (4.1)	2 (2.5)	
	Others	51 (30.0)	15 (26.3)		41 (28.1)	25 (30.9)	
Monthly income	< 20,000 BDT (< $163.93)	78 (45.9)	24 (42.1)	0.784	67 (45.9)	35 (43.2)	0.896
	20,000–40,000 BDT (163.93–327.86 USD)	57 (33.5)	22 (38.6)		49 (33.6)	30 (37.8)	
	> 40,000 BDT (> $327.86)	35 (20.6)	11 (19.3)		30 (20.5)	16 (19.8)	
Type of family	Nuclear	105 (61.8)	30 (52.6)	0.224	87 (59.6)	48 (59.3)	0.961
	Joint	65 (38.2)	27 (47.4)		59 (40.4)	33 (40.7)	
Place of residence	Urban	136 (80.0)	49 (86.0)	0.316	119 (81.5)	66 (81.5)	0.996
	Rural	34 (20.0)	8 (14.0)		27 (18.5)	15 (18.5)	
Gestational age (in weeks)	First trimester (0–13 weeks)	47 (27.6)	19 (33.3)	0.478	34 (23.3)	32 (39.5)	**0.025**
	Second trimester (14–27 weeks)	64 (37.6)	23 (40.4)		58 (39.7)	29 (35.8)	
	Third trimester (28–42 weeks)	59 (34.7)	15 (26.3)		54 (36)	20 (24.7)	
BMI	Under weight	16 (9.5)	5 (8.8)	0.162	13 (9)	8 (10)	0.683
	Healthy weight	102 (60.7)	40 (70.2)		92 (63.4)	50 (62.5)	
	Overweight	33 (19.6)	9 (15.8)		25 (17.2)	17 (21.2)	
	Obese	17 (10.1)	3 (5.3)		15 (10.3)	5 (6.3)	
Anemia	Yes	38 (22.4)	18 (31.6)	0.533	33 (22.6)	23 (28.4)	0.332
	No	132 (77.6)	39 (68.4)		113 (77.4)	58 (71.6)	
Relationship with husband	Good	169 (99.4)	50 (87.7)	**< 0.001**	142 (97.3)	77 (95.1)	0.371
	In‐between	1 (0.6)	6 (10.5)		4 (2.7)	3 (3.7)	
	Bad	0 (0)	1 (1.8)		0 (0)	1 (1.2)	
Relationship with mother‐in‐law	Good	144 (84.7)	43 (75.4)	0.170	125 (85.6)	62 (76.5)	0.385
	In‐between	3 (1.8)	4 (7)		4 (2.7)	3 (3.7)	
	Bad	5 (2.9)	3 (5.3)		4 (2.7)	4 (4.9)	
	Not applicable (e.g., if dead)	18 (10.6)	7 (12.3)		13 (8.9)	12 (14.8)	
Receive help in daily activities	Yes	148 (87.1)	50 (87.7)	0.897	119 (81.5)	63 (77.8)	0.500
	No	22 (12.9)	7 (12.3)		27 (18.5)	18 (22.2)	
Faced physical violence	Yes	24 (14.1)	14 (24.6)	0.068	21 (14.4)	17 (21)	0.202
	No	146 (85.9)	43 (75.4)		125 (85.6)	64 (79)	
Faced physical violence during pregnancy	Yes	5 (2.9)	4 (7)	0.172	4 (2.7)	5 (6.2)	0.204
	No	165 (170)	53 (93)		142 (93.3)	76 (93.8)	
Faced forced sex	Yes	3 (1.8)	5 (8.8)	**0.013**	6 (4.1)	2 (2.5)	0.521
	No	167 (98.2)	52 (91.2)		140 (95.9)	79 (97.5)	
Parity	Primipara	67 (39.4)	27 (48.2)	0.246	60 (41.1)	34 (42.5)	0.838
	multipara	103 (60.6)	29(51.8)		86 (58.9)	46 (57.5)	
History of abortion	Yes	20 (11.8)	10 (17.5)	0.272	17 (11.6)	13 (16.3)	0.329
	No	149 (88.2)	47 (82.5)		129 (88.4)	67 (83.8)	
Planed pregnancy	Yes	136 (81.0)	43 (75.4)	0.327	120 (83.3)	59 (72.8)	0.061
	No	32 (19.0)	14 (24.6)		24 (16.7)	22.7 (81)	

*Note:* Bold values are statistically significant at *p* < 0.05.

### 5.5 Risk Factors of Depression and Anxiety

The results of multiple logistic regression analysis to determine the risk factors associated with depression during pregnancy are shown in Table 4. Among socio‐demographic, physiological condition, family support, husbands' violence, and obstetric profiles, two factors have a significant association with depression (*p* < 0.05). Housewives showed a significantly lower likelihood of prenatal depression [AOR = 0.17, 95% CI: 0.05–0.55, *p*‐value = 0.003] compared to working women. Conversely, working women were 5.88 times more likely to suffer from prenatal depression. Moreover, the women who experienced forceful sex were 4.39 times more likely to have depression compared to the women who had not (AOR = 4.39, 95% CI: 0.97–19.82, *p*‐value = 0.054). Although the *p*‐value is marginally above the conventional threshold of significance, it suggests a strong potential association warranting further exploration.

**Table 4:** Multiple logistic regression analysis showing associated factors influencing depression during pregnancy.

Variable	Categories	AOR (95% CI)	*p*‐value
Age	≤ 18	0.12 (0.01–1.39)	0.091
	19–34	0.14 (0.03–0.83)	
	≥ 35	Ref.	
Educational qualification	Illiterate	0.39 (0.02–6.48)	0.884
	Primary	0.72 (0.26–2.01)	
	Secondary	0.88 (0.35–2.19)	
	Higher	Ref.	
Husband's educational qualification	Illiterate	0.88 (0.18–4.3)	0.893
	Primary	0.71 (0.25–2.03)	
	Secondary	1.04 (0.42–2.71)	
	Higher	Ref.	
Occupation	Housewife	0.17 (0.05–0.55)	0.003
	Working women	Ref.	
Monthly income	< 20,000 BDT (< $163.93)	0.40 (1.59–0.53)	0.571
	20,000–40,000 BDT ($163.93–327.86)	0.29 (1.72–0.62)	
	> 40,000 BDT (> $327.86)	Ref.	
Type of family	Nuclear	0.55(0.27–1.12)	0.102
	Joint	Ref.	
Place of residence	Urban	1.35 (0.55–3.30)	0.500
	Rural	Ref.	
Gestational age (in weeks)	First trimester (0–13 weeks)	1.69(0.761–3.77)	0.421
	Second trimester (14–27 weeks)	1.43 (0.67–3.06)	
	Third trimester (28–42 weeks)	Ref.	
BMI	Underweight	1.53 (0.30–7.67)	0.616
	Healthy weight	2.094 (0.57–7.63)	
	Overweight	1.51 (0.36–6.39)	
	Obese	Ref.	
Anemia	Yes	1.65 (0.84–3.25)	0.146
	No	Ref.	
Relationship with mother‐in‐law	Good	0.74 (0.289–1.92)	0.203
	In‐between	3.36 (0.594–19.10)	
	Bad	1.62 (0.298– 8.891)	
	Not applicable (e.g., if dead)	Ref.	
Received help in daily activities	Yes	1.22 (0.47–3.20)	0.674
	No	Ref.	
Faced physical violence	Yes	1.66 (0.71–3.91	0.241
	No	Ref.	
Faced physical violence during pregnancy	Yes	1.22 (0.24–6.03	0.802
	No	Ref.	
Faced forced sex	Yes	4.39 (0.97–19.82)	0.0542
	No	Ref.	
Parity	Primipara	1.75 (0.91–3.37)	0.091
	Multipara	Ref.	
History of abortion	Yes	2.048 (0.851– 4932)	0.110
	No	Ref.	
Planned pregnancy	Yes	0.60 (0.27–1.35)	0.605
	No	Ref.	

Abbreviations: AOR = adjusted odds ratio, CI = confidence interval, Ref. = reference category.

Table 5 highlights the multiple logistic regression analysis of factors associated with anxiety during pregnancy. Gestational age showed a significant association with anxiety during pregnancy. Women in the first trimester (0–13 weeks) were 2.63 times more likely to experience anxiety compared to those in the third trimester (AOR = 2.63, 95% CI: 1.27–5.45, *p*‐value = 0.028). Similarly, women in their second trimester showed a 1.42 times higher likelihood of experiencing anxiety compared to those in the third trimester (AOR = 1.42, 95% CI: 0.71–2.84, *p*‐value = 0.028). Lastly, we haven't found any significant association between BMI and prenatal anxiety.

**Table 5:** Multiple logistic regression analysis showing associated factors influencing Anxiety during pregnancy.

Variable	Categories	AOR (95% CI)	*p*‐value
Age	≤ 18	1.52 (0.17– 13.30)	0.653
	19–34	0.79 (0.16–3.96)	
	≥ 35	Ref.	
Educational qualifications	Illiterate	0.00 (0.00–0.00)	0.503
	Primary	0.75 (0.31–1.81)	
	Secondary	1.42 (0.65–3.14)	
	Higher	Ref.	
Husband's educational qualification	Illiterate	0.79 (0.19–3.17)	0.198
	Primary	0.37 (0.15–0.94)	
	Secondary	0.67 (0.29–1.54)	
	Higher	Ref.	
Occupation	Housewife	0.65 (0.29–1.54)	0.428
	Working women	Ref.	
Monthly income	< 20,000 BDT (< $163.93)	1.84 (0.73–4.65)	0.388
	20,000–40,000 BDT ($163.93–$327.86)	1.71 (0.73–4.01)	
	> 40,000 BDT (> $327.86)	Ref.	
Type of family	Nuclear	1.21 (0.65–2.29)	0.54
	Joint	Ref.	
Place of residence	Urban	0.91 (0.43–1.92)	0.804
	Rural	Ref.	
Gestational age (in weeks)	First trimester (0–13 weeks)	2.63 (1.27–5.45)	0.029
	Second trimester (14–27 weeks)	1.42 (0.71–2.84)	
	Third trimester (28–42 weeks)	Ref.	
BMI	Under weight	1.51 (0.38–5.98)	0.686
	Healthy weight	1.53 (0.51–4.57)	
	Overweight	2.07 (0.62–6.94)	
	Obese	Ref.	
Anemia	Yes	1.48 (0.78–2.81)	0.224
	No	Ref.	
Parity	Primipara	1.18 (0.65–2.14)	0.582
	Multipara	Ref.	
History of abortion	Yes	0.58 (0.29–1.15)	0.120
	No	Ref.	
Planed pregnancy	Yes	1.50 (0.66–3.39)	0.328
	No	Ref.	
Relationship with mother‐in‐law	Good	0.54 (0.23–1.28)	0.546
	In‐between	0.82 (0.14–4.61)	
	Bad	0.80 (0.14–4.52)	
	Not applicable (e.g., if dead)	Ref.	
Receive help with daily activities	Yes	0.922 (0.38–2.01)	0.850
	No	Ref.	
Faced physical violence	Yes	1.43 (0.64–3.19)	0.382
	No	Ref.	
Faced physical violence during pregnancy	Yes	2.03 (0.42–9.62)	0.371
	No	Ref.	
Faced forced sex	Yes	0.43 (0.08–2.38)	0.333
	No	Ref.	

Abbreviations: AOR = adjusted odds ratio, CI = confidence interval, Ref. = reference category.

## 6 Discussion

This study investigated the prevalence of depression and anxiety during pregnancy and the factors associated with them among pregnant women in Bangladesh. Various pregnancy‐related risk factors were analyzed, including sociodemographic characteristics, obstetric history, family support, physical and sexual abuse by their husbands, and physiological measurements such as BMI and presence of anemia.

The study reported a prevalence of depression of 25.1% (using an EPDS cutoff score ≥ 10), which is higher than the prevalence of Middle Eastern (19.5%), Western Europeans (8.6%), and South Asians (17.5%) found in a systematic assessment [39]. However, the prevalence of depression in this study shows similar findings to some previous studies from low‐ and middle‐income nations. For instance, the prevalence of depression was 27% in Pakistan [40], 28% in India (EPDS score of ≥ 13) [41], and 22.4% in Nigeria (EPDS score of > 10) [42]. Interestingly, the prevalence of depression in the antenatal period in Canada also showed a nearly similar prevalence of 27% [43]. An earlier study in Bangladesh indicated a greater prevalence of depression during pregnancy (33%), which may be due to the collection of data of women who belong only in the third trimester, unlike the broader approach used in this study [44]. The stage of pregnancy over which indicators are examined, the sorts of instruments employed, such as traditional clinical surveys or screening tools, and various cut‐off points on screening tools can all have an impact on the frequency of depression [20].

However, a lower prevalence (18%) of depression during pregnancy was reported in Bangladesh [20]. This discrepancy could be explained by the variance in socioeconomic level, sociocultural variation, and psychosocial elements like support networks that may fluctuate across the nation's many areas [45]. The present study and those two studies were different regarding sociodemographic setting and sample size. Another Bangladeshi study reported an 18.3% prevalence of depression among pregnant women who were attending the Bangladesh Institute of Research and Rehabilitation in Diabetes, Endocrine and Metabolic Disorders [46].

This study found the prevalence of anxiety during pregnancy was 35.7% (using an STAI cutoff score ≥ 45), which is higher than the previous Bangladeshi study that reported the prevalence of antenatal anxiety as 29% [20]. Similarly, a lower prevalence of pregnancy‐related anxiety was reported among the women of Saudi Arabia [47]. They reported about 23.6% pregnancy‐related anxiety, whereas Indian women reported more anxiety (55.7%) [48]. In Brazil, 26.8% of pregnant women suffered from pregnancy‐related anxiety [49], and in South Africa, the prevalence was 23% [50].

The present study found that women of their gestational age (first trimester) had higher anxiety. Similar findings have been asserted elsewhere. That study depicted women in their first, second, and third trimesters having 9%, 13%, and 21% severe anxiety levels, respectively [51]. The study also reported an elevated level of anxiety in women of the third trimester compared to those in the first trimester of their gestational age, which is contradictory to our present findings reporting that women in their first trimester were more anxious.

Antenatal depression's causative factors or pathophysiology are still inadequately known. It was proposed that depression at this time may be linked to hormonal changes connected to pregnancy, birth, and cognitive function [52]. The connection between poverty and depression has been extensively established, and evidence from low‐income nations also supports this connection [53].

The study demonstrated that participants' occupational status was significantly associated with depression. More specifically, working women were at a 5.88 times higher risk of depression compared to housewives. Similarly, a previous study in Taiwan reported that working women demonstrated a higher risk of depression during their pregnancy due to several factors, such as job stress, poor sleep status, and a lack of help from family or friends, especially in the third trimester of pregnancy and after returning to work [54]. Another study showed that 76.1% of working women present major depressive symptoms during their pregnancy period [55]. Several factors, like strange work shifts, higher work stress, and employment in the medical care and technology sectors, increase the risk of developing prenatal depression [55]. However, opposite findings have been reported elsewhere [56]. They reported that despite having an education, not working outside the home could increase the risk of depression during pregnancy, according to a study in Bangalore [56]. Studies from low‐income settings indicated that housewives were more likely to have depressive symptoms compared with other groups during pregnancy [45]. This difference in results might have happened due to the job experience, work strains, and the roles of housewives were different among different countries. A qualitative study should be conducted to find out more underlying factors related to prenatal depression. This study also reported a significant association between forceful sex and depression among pregnant women. About 16.7% of pregnant women in this study experienced violence from their partner, and 3.5% faced forced sex. Physical abuse by a husband of the woman during pregnancy was an important risk factor for antenatal depression, which is compatible with earlier studies carried out in both industrialized and developing nations [57].

The findings of this study highlight the significant prevalence of prenatal depression and anxiety among pregnant women in Bangladesh. It has been clearly identified that occupational status, intimate partner violence, and gestational age are key risk factors. These results emphasize the need for integrated mental health screening in antenatal care, especially for at‐risk groups such as working women and those experiencing intimate partner violence. Public health interventions should focus on increasing awareness, reducing stigma, and providing targeted support for women facing socioeconomic hardships. Moreover, policies addressing workplace stress and access to mental health services during antenatal care are crucial. Further research is needed to explore the underlying cultural and socio‐demographic factors that contribute to maternal mental health issues, guiding the development of more effective, context‐specific interventions.

## 7 Strengths and Limitations

This study highlights a significant public health concern by exploring the prevalence of anxiety and depression among pregnant women in Bangladesh, a low‐ and middle‐income country. The study recruited 227 participants, providing a substantial data set for analysis. Additionally, the comprehensive questionnaire addressed socio‐demographic factors, physiological conditions, family support, intimate partner violence, and obstetric history, offering a multifaceted perspective on prenatal mental health. Furthermore, validated instruments such as the Edinburgh Postnatal Depression Scale (EPDS) and the State‐Trait Anxiety Inventory (STAI) were utilized, ensuring reliable and valid assessments. The study′s findings reveal a high prevalence of prenatal depression (25.1%) and anxiety (35.7%), underscoring the urgent need for policy interventions to address these issues.

This study focused on antenatal depression, which has received less focus than postnatal depression. This study had some limitations. This was a hospital‐based cross‐sectional study conducted in a government hospital. So, this study did not include pregnant women who were accessing private healthcare or those not seeking hospital care. Furthermore, the cross‐sectional design of the study limits its ability to establish causal relationships between the variables. Furthermore, this study relied on self‐reported measures to assess symptoms of anxiety and depression. While these tools are validated for screening, they do not provide a clinical diagnosis. We do not consider that participants' or partners' healthcare professions may influence knowledge levels and health‐seeking behavior. Lastly, the monocentric design limits the representativeness of the sample, as sociocultural and socioeconomic factors may vary across different regions of Bangladesh. Future studies should consider multi‐center designs to capture a broader range of experiences from different geographical and socioeconomic settings.

## 8 Recommendations

This study emphasizes the urgent need to incorporate mental health screening into routine antenatal care in Bangladesh. In antenatal visits, routine psychological assessments should be included, particularly during the first and second trimesters. Additionally, targeted interventions are necessary for high‐risk groups, such as working women and those who have experienced intimate partner violence. There should be training sessions arranged for healthcare providers to recognize and manage mental health concerns. Furthermore, community awareness campaigns and collaboration with local NGOs and health workers can enhance mental health literacy and encourage early help‐seeking. Policymakers should prioritize maternal mental health by strengthening guidelines, investing in mental health infrastructure, and ensuring that services are available across both the public and private sectors.

## 9 Conclusions

This study found a high prevalence of prenatal depression (25.1%) and anxiety among pregnant women (35.7%). Occupation, forceful sex, and gestational age are significantly associated with depression and anxiety. The likelihood of having a lower risk of depression among pregnant women who were housewives, compared to working women. However, aggressive sex increased the risk of depression among pregnant women. The study also found that women in their first and second trimesters were suffering from more mental health problems than those who were in their third trimester. Therefore, special attention should be given to that cohort, considering the severity of mental health symptoms. Enhancing the psychological well‐being of expectant mothers can be facilitated by government health care facilities, women′s groups, and local development organizations.

## Author Contributions

**Noushin Rahman Mahin:** conceptualization, formal analysis, writing – review and editing, writing – original draft, investigation, data curation. **Md Jamil Hossain:** conceptualization, methodology, writing – original draft, writing – review and editing, formal analysis, investigation. **Bishwjit Bhowmick:** writing – review and editing, formal analysis, investigation. **Lakshmi Rani Kundu:** conceptualization, investigation, writing – review and editing, writing – original draft, formal analysis, supervision, validation.

## Ethics Statement

The study received approval from the Biosafety, Biosecurity, and Ethical Review Board of Jahangirnagar University, Savar, Dhaka‐1342, Bangladesh (Ref. No: BBEC, JU/M 2024/08 (123)). Permission was obtained from the authority of the Institute of Child and Mother Health, Matuail, Dhaka, before data collection. Participants were informed about the nature and purpose of the study and the confidentiality of their data. They had the right to withdraw from the study at any time during the interview. Additionally, informed written consent was obtained from all participants.

## Conflicts of Interest

The authors declare no conflicts of interest.

## Transparency Statement

The lead author, Noushin Rahman Mahin, affirms that this manuscript is an honest, accurate, and transparent account of the study being reported; that no important aspects of the study have been omitted; and that any discrepancies from the study as planned (and, if relevant, registered) have been explained.

## Data Availability

The data set was not publicly available, but it would be accessible from the corresponding author upon reasonable request.
